# BluePort: A Platform to Study the Eosinophilic Response of Mice to the Bite of a Vector of *Leishmania* Parasites, *Lutzomyia longipalpis* Sand Flies

**DOI:** 10.1371/journal.pone.0013546

**Published:** 2010-10-27

**Authors:** J. Santiago Mejia, Amanda L. Toot-Zimmer, Patricia C. Schultheiss, Barry J. Beaty, Richard G. Titus

**Affiliations:** 1 Department of Microbiology, Immunology and Pathology, Colorado State University, Fort Collins, Colorado, United States of America; 2 MicroRx Infectious Disease Supercluster, Colorado State University, Fort Collins, Colorado, United States of America; New York University School of Medicine, United States of America

## Abstract

**Background:**

Visceral Leishmaniasis is a serious human disease transmitted, in the New World, by *Lutzomyia longipalpis* sand flies. Natural resistance to *Leishmania* transmission in residents of endemic areas has been attributed to the acquisition of immunity to sand fly salivary proteins. One theoretical way to accelerate the acquisition of this immunity is to increase the density of antigen-presenting cells at the sand fly bite site. Here we describe a novel tissue platform that can be used for this purpose.

**Methodology/Principal Findings:**

BluePort is a well-vascularized and macrophage-rich compartment induced in the subcutaneous tissue of mice via injection of agarose beads covered with Cibacron blue. We describe the sequence of inflammatory events leading to its formation and how it can be used to study the dermal response to the bite of *L. longipalpis* sand flies. Results presented indicate that a shift in the inflammatory response, from neutrophilic to eosinophilic, is the main histopathological feature associated with the immunity acquired through repeated exposure to the bite of sand flies, and that the BluePort tissue compartment could be used to accelerate this process. In addition, changes observed inside the BluePort parenchyma indicate that it could be used to study complex immunobiological processes, and to develop ectopic secondary lymphoid structures.

**Conclusions/Significance:**

Understanding the characteristics of the dermal response to the bite of sand flies is a critical element of strategies to control leishmaniasis using vaccines that target salivary proteins. Finding that dermal eosinophilia is such a prominent component of the anti-salivary immunity induced by repeated exposure to sand fly bites raises one important consideration: how to avoid the immunological conflict derived from a protective Th2-driven immunity directed to sand fly saliva with a protective Th1-driven immunity directed to the parasite. The BluePort platform is an ideal tool to address experimentally this conundrum.

## Introduction

Leishmaniasis is a group of parasitic diseases transmitted to humans and animals through the bite of phlebotomine sand flies infected with parasitic protozoans of the genus *Leishmania* [1], [2], [3]. The wide variety of clinical presentations of these diseases [4], [5], [6], [7], [8] is a reflection of the numerous host-, parasite- and vector-derived factors playing a role in their pathogenesis [9], [10], [11], [12], [13], [14], [15], [16], [17], [18], [19]. Among these factors, the infection-potentiating effect of sand fly salivary molecules [17], [18], [19], [20] has generated a great deal of excitement in the research community because: 1) it provides clues about immunobiological determinants of resistance or susceptibility to *Leishmania* infection and 2) it provides additional targets for vaccines to prevent leishmaniasis. Sand fly saliva plays an important role in the transmission of *Leishmania* parasites, facilitating their survival and dissemination in tissues of the vertebrate hosts by promoting a Th2-skewed immune response at the bite site [21], [22], [23]. Vaccines directed to sand fly saliva are expected to induce protective immunity by neutralizing the biological activity of salivary immuno-modulators and by generating a tissue microenvironment that promotes the destruction of parasites delivered, along with saliva, while sand flies take a blood meal [17], [24], [25]. Epidemiological evidence linking resistance to *Leishmania* infection in adults living in endemic areas with production of antibodies to sand fly salivary antigens, indicates that protective anti-salivary immunity can be acquired through chronic exposure to the bite of sand flies [26], [27]. Given that arthropod saliva is a cocktail of molecules selected through evolution to optimize access to the blood of vertebrates and minimize immune reactions [17], [20], [28], [29], it is not surprising that anti-salivary immunity takes so long to develop under natural conditions. The fate of arthropod-salivary proteins delivered at the bite site is an additional factor that might determine the speed at which the vertebrate host acquires protective anti-salivary immunity. This is because neutrophils, one of the major components of the wound resolution machinery of vertebrates [30], can degrade arthropod salivary proteins before they are taken up by professional antigen-presenting cells. Histopathological analysis of sand fly bite sites indicates that neutrophils are indeed a dominant component of the early inflammatory response to sand fly bites in naïve animals [31], [32]. Theoretically, the acquisition of natural immunity to sand fly saliva would be accelerated if changes introduced into vertebrate tissues decrease the influx of neutrophils to the bite site or, alternatively, improve access of professional antigen-presenting cells to salivary proteins before they are degraded by neutrophil-derived enzymes. The former can be induced with drugs or anti-neutrophil antibodies [33], [34], but the associated systemic vulnerability to bacterial infections is a major drawback of this approach. The latter, on the other hand, can be limited to small skin areas to minimize unintended adverse side effects. During experimental evaluation of mechanisms to increase the density of professional antigen-presenting cells in the subcutaneous tissue of mice, we found that a well-vascularized and stable tissue compartment enriched in macrophages can be induced by the injection of agarose beads covered with the triazine dye Cibacron Blue. Here we describe the characteristics of this tissue compartment (BluePort), the sequence of inflammatory events leading to its formation, and how it can be used to study the dermal response to the bite of *Lutzomyia longipalpis* sand flies.

## Results

### Induction of BluePort formation

**Figure 1:**
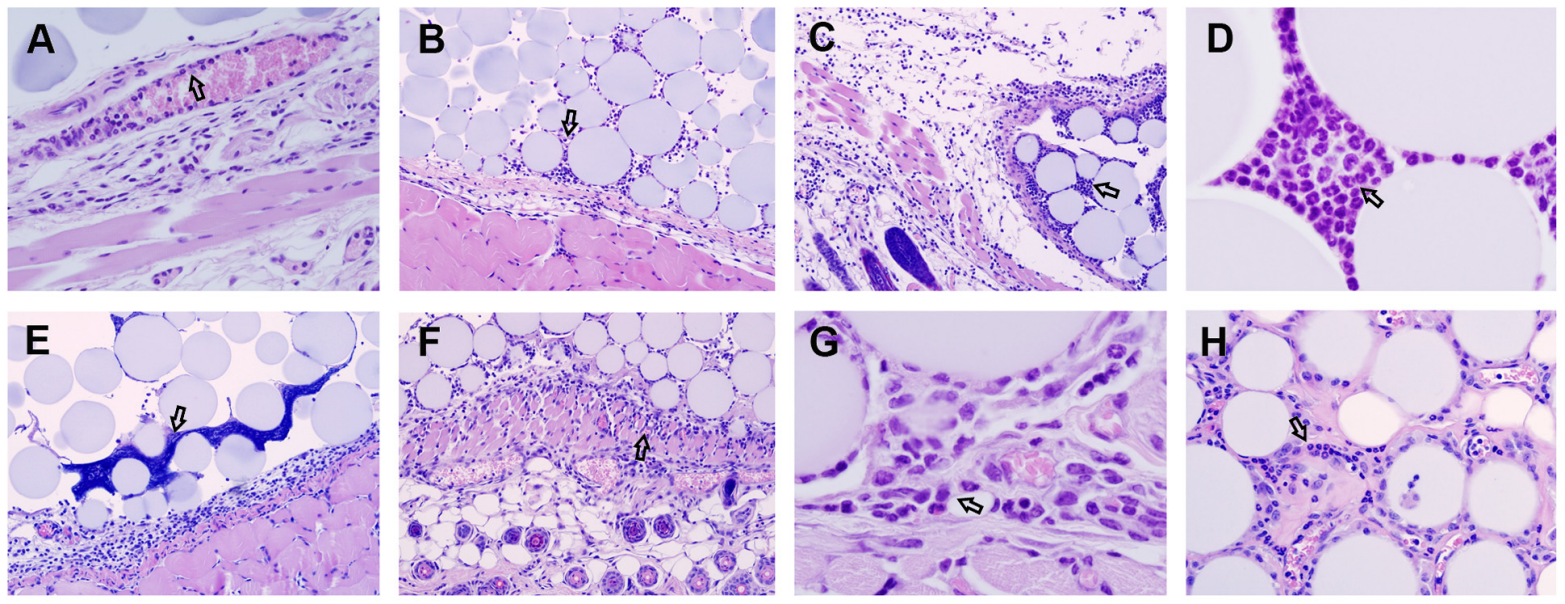
Acute inflammatory response to CBa-beads. Histopathological changes observed 6 hours (A and B), 24 hours (C and D), 48 hours (E), 96 hours (F and G) and 15 days (H) after injection of CBa-beads in the subcutaneous tissue of BALB/c mice. Arrows indicate marginalized neutrophils in (A), neutrophils in the space between CBa-beads in (B, C and D), neutrophil extracellular traps (NETs) in (E), and mixed cellular infiltrate in (F-H). Hematoxylin-eosin stain. Magnification: 200x (B, C and E), 400x (A and H) and 1000x (D and G).

**Figure 2:**
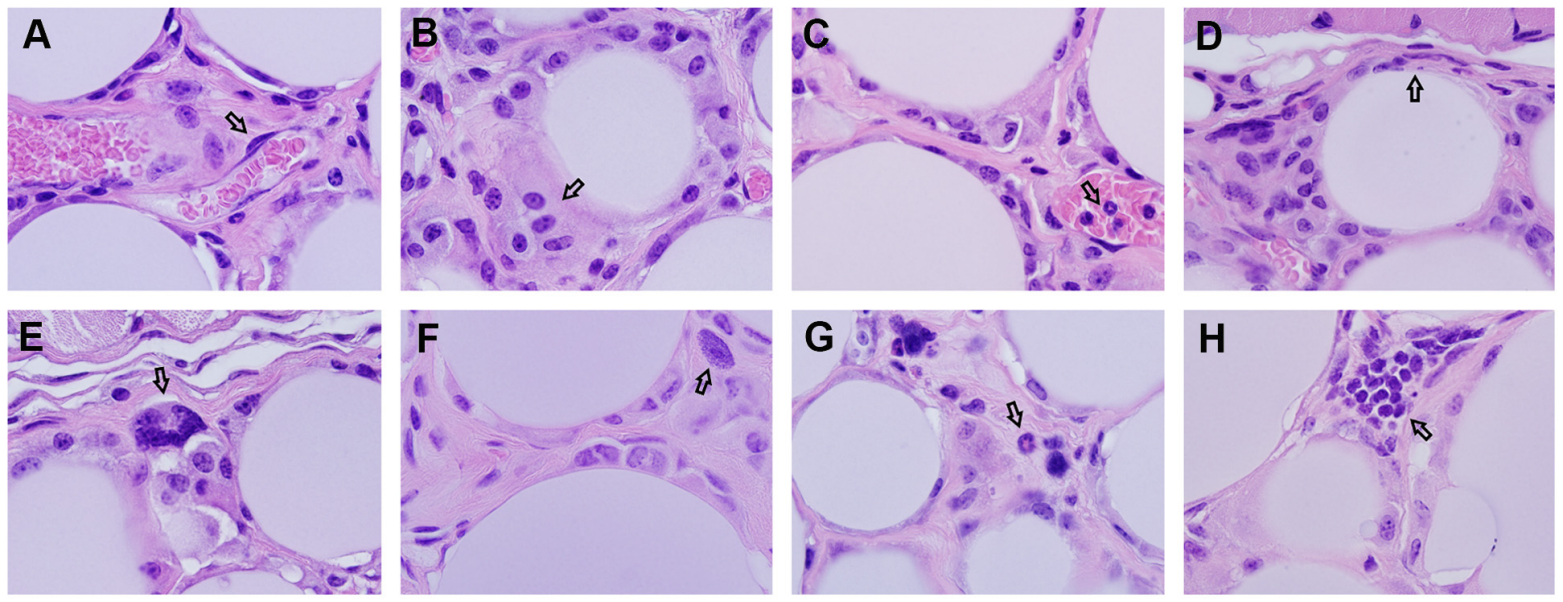
Chronic inflammatory response to CBa-beads. Histopathological changes observed 30 days (A and B), 60 days (C and D), 90 days (E and F) and 120 days (G and H) days after injection of CBa-beads. Arrows indicate endothelial cells in (A), macrophages in (B), neutrophils inside a blood vessel in (C), capsule in (D), multinucleated foreign-body giant cells in (E), mast cells in (F), eosinophils in (G) and lymphocytes in (H). Hematoxylin-eosin stain, 1000x magnification.

**Figure 3:**
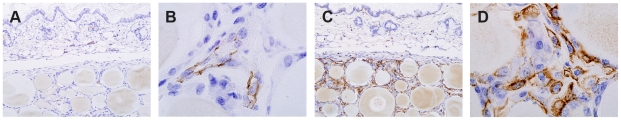
Immuno-histochemical analysis of BluePort-resident cells. Reactivity of antibodies to CD31, marker of endothelial cells, in (A and B), and to F4/80, marker of macrophages, in (C and D). Magnification 200x (A and C) and 1000x (B and D).

Cibacron blue-agarose (CBa) beads injected in the subcutaneous tissue of mice remain in place without evidence of degradation or tissue rejection for up to 4 months. Upon inspection at the microscopic level, a typical acute inflammatory reaction developed at the site of injection with edema, vasodilatation of dermal blood vessels, marginalization and migration of neutrophils into the space between the beads. This was initially detected 6 hours post-injection (Figure 1A and B) and continued until the space between the beads was found replete with neutrophils 24 hours post-injection (Figure 1C and D). A basophilic amorphous material, likely to represent neutrophil extracellular traps (NETs) [35], was found between the beads 48 hours post-injection (Figure 1E). The influx of neutrophils waned afterwards and a mixed infiltrate of neutrophils, eosinophils and mononuclear cells was found at the interface between the beads and mouse tissues 96 hours post-injection (Figures 1F and G). By the second week following the injection of CBa-beads only a few neutrophils were found infiltrating a space occupied mostly by mononuclear cells (Figures 1H). In samples taken 30 and 60 days post-injection, it was found that the CBa-beads were integrated into a well-vascularized tissue compartment enriched in macrophages and surrounded by a thin fibrous capsule (Figure 2A–D). The beads did not appear damaged and only a few foreign-body multinucleated giant cells were found (Figure 2E), an indication that the beads were no longer generating danger signals to the innate immune system. In support of this interpretation was the finding of collagen-rich extracellular matrix deposition, a terminal event of the wound healing and tissue regeneration process [36], in scattered areas within the bead-generated compartment 90 and 120 days post-injection (Figure 2F–H). Mast cells, eosinophils and rarely lymphocytes, were found in some of these collagen-rich areas (Figure 2F–H). Immuno-histochemical analysis confirms the phenotype of the two most abundant cells in the BluePort parenchyma, endothelial cells (CD31+) (Figure 3A and B) and macrophages (F4/80+) (Figure 3C and D).

### BluePort-associated skin to study tissue response to sand fly bites in naïve and immune mice

**Figure 4:**
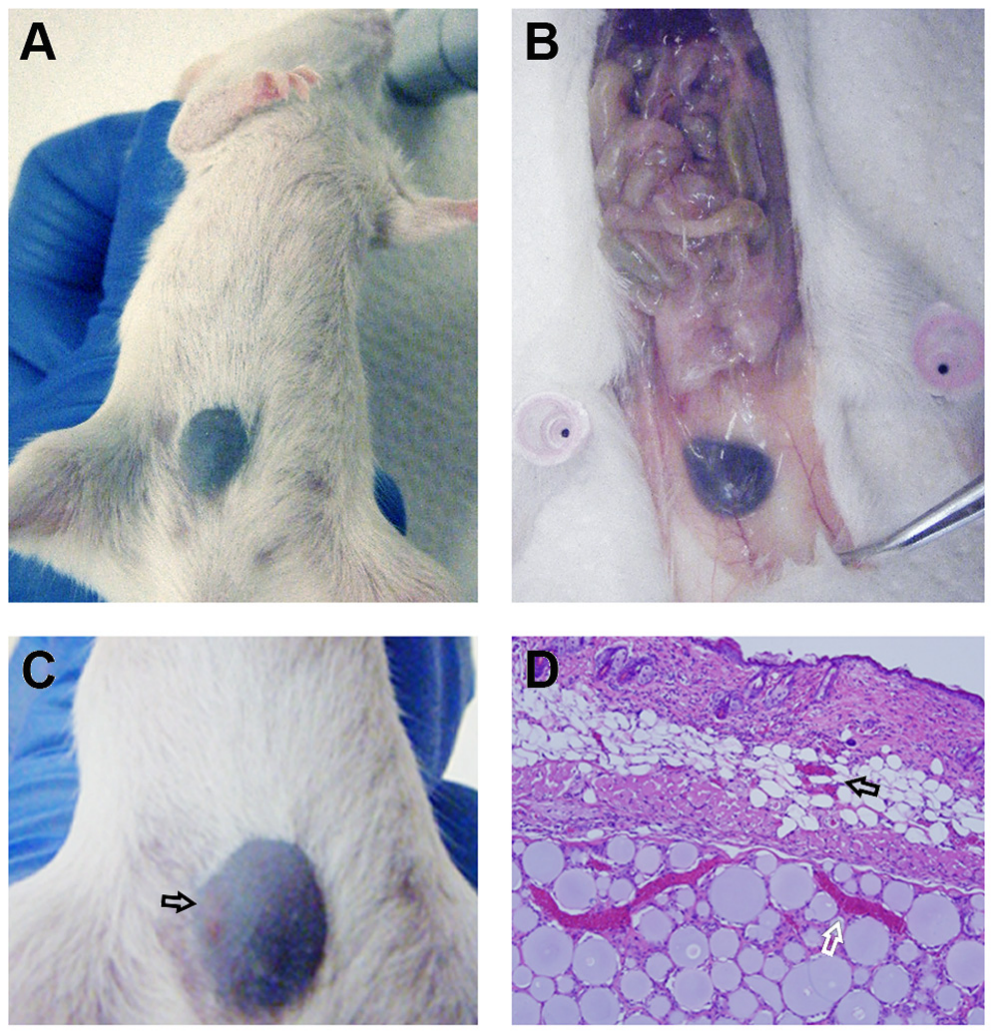
Localization and appearance of the BluePort tissue compartment. Macroscopic appearance of the blue nodule (BluePort) formed one month after injection of CBa-beads in the subcutaneous tissue of mice (A and B). Macroscopic (C) and microscopic appearance (D) of BluePort-associated skin 24 hours after exposure of naïve mice to the bite of *L. longipalpis* sand flies. Black arrows indicate skin erythema in (C), and vasodilatation of dermal blood vessels in (D). White arrow indicates vasodilatation of blood vessels inside the BluePort parenchyma in (D). Hematoxylin-eosin stain, magnification 100x in (D).

**Figure 5:**
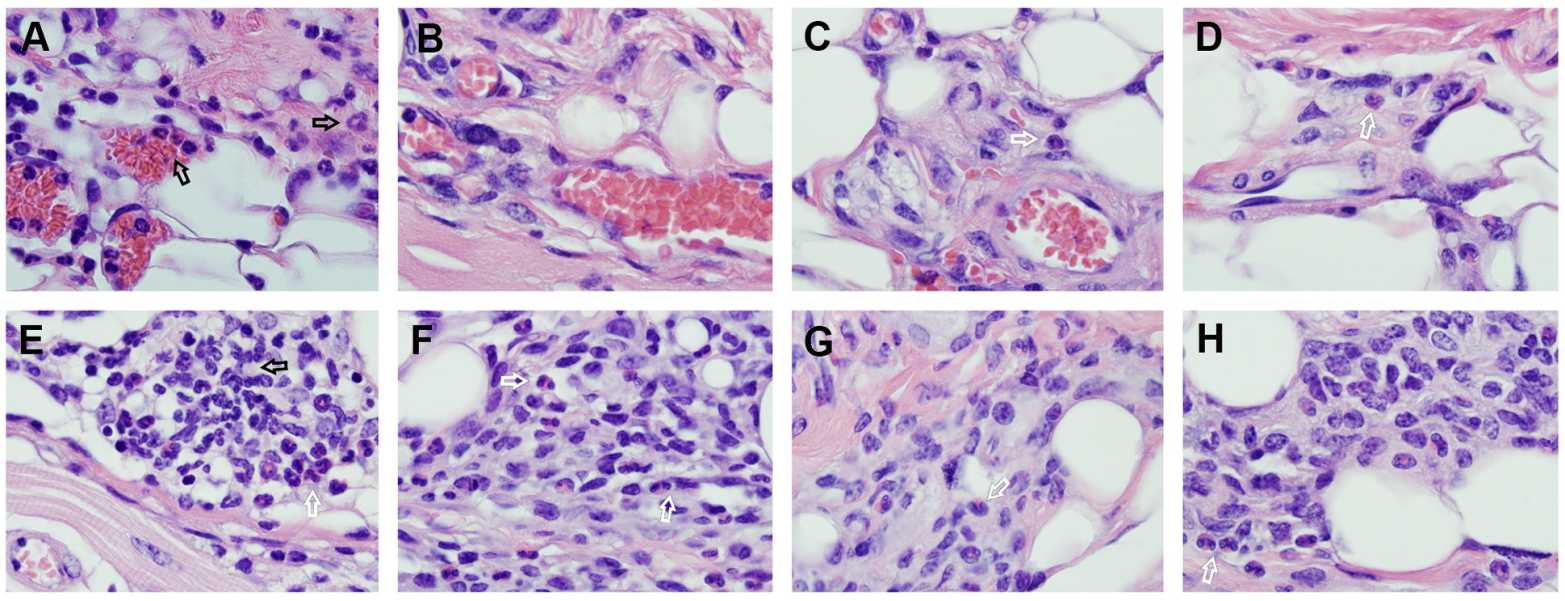
Inflammatory response of naïve and immune mice to sand fly bites on BluePort-associated skin. Evolution of the inflammatory response on naïve mice (A–D), and mice pre-exposed multiple times to the bite of sand flies (E–H). Images correspond to samples taken 24 hours (A and E), 48 hours (B and F), 72 hours (C and G) and 96 hours (D and H) post-exposure. Black arrows indicate neutrophils and white arrows eosinophils. Hematoxylin-eosin stain, 1000x magnification.

**Figure 6:**
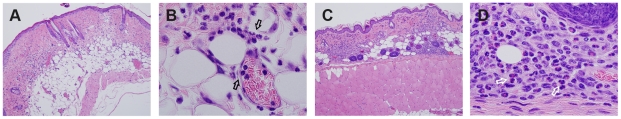
Inflammatory response of naïve and immune mice to sand fly bites on normal skin. Inflammatory response detected 48 hours after exposure to the bite of *L. longipalpis* sand flies on naïve mice (A and B) and immune mice (C and D). Black arrows indicate neutrophils and white arrows eosinophils. Hematoxylin-eosin stain. Magnification, 200x in (A and C), 1000X in (B and D).

**Figure 7:**
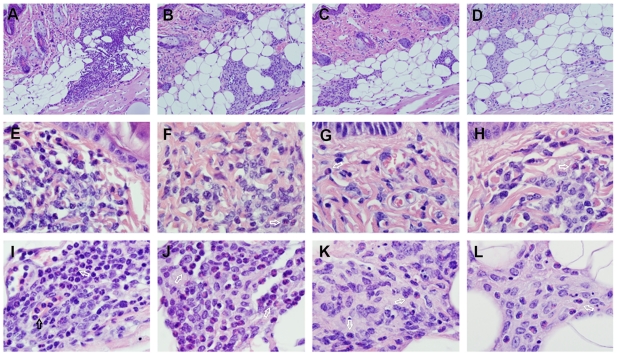
Inflammatory response in mice exposed twice to sand fly bites on BluePort-associated skin. Evolution of the inflammatory response in samples collected 24 hours (A, E and I), 48 hours (B, F and J), 72 hours (C, G and K) and 96 hours (D, H and L) after exposure for the second time to the bite of sand flies. Black arrows indicate neutrophils and white arrows indicate eosinophils. Hematoxylin-eosin stain. Magnification: 100x in (A–D), 1000x in dermis (E–H) and hypodermis (I–L).

The blue nodule (BluePort) formed in the subcutaneous tissue of mice one month after injection of CBa-beads rests under a normal looking skin (Figure 4A and B) that, after shaving, can be used as an access window for sand flies to obtain a blood meal. Adult *L. longipalpis* female sand flies successfully blood-feed on this skin area, causing one of the well known clinical features of sand fly bites, intense erythema that last several hours [27] (Figure 4C). This erythema is associated, at the microscopic level, with a strong vasodilatory response of dermal blood vessels (Figure 4D). A concomitant vasodilatory response of vessels irrigating the BluePort parenchyma indicates that these new vessels respond to vasodilatory signals generated at the bite site, acting as a functional unit with the adjacent dermal vessels (Figure 4D). In addition to the vascular response, edema, marginalization and infiltration of the dermis by neutrophils were the main characteristics of the tissue response to the bite of sand flies on BluePort-associated skin of naïve mice (Figures 5A–D). These features were prominently expressed 24 hours after exposure and progressively decreased afterwards with few traces of inflammation, including the presence of few eosinophils, 72 and 96 hours post-exposure (Figure 5C and D). In contrast to the mild and transitory neutrophilic inflammatory reaction of naïve mice to the bite of sand flies, the inflammatory reaction in mice pre-exposed multiple times to the bite of sand flies was characterized by intense and protracted infiltration of dermis and hypodermis by eosinophils and mononuclear cells (Figure 5 E–H). This change from a predominantly neutrophilic infiltrate to a predominantly eosinophilic infiltrate does not seem to be attributed to an effect mediated by the BluePort because a similar shift in granulocyte dominance was observed when exposure to the bite of sand flies occurred on normal skin (Figure 6). This neutrophil-to-eosinophil shift was also found in samples taken from mice in which *L. longipalpis* sand flies were allowed to take a blood-meal for a second time, one month after the first exposure on BluePort-associated skin (Figure 7). While some neutrophils were found in the hypodermis of these mice 24 hours after the second exposure to the bite of sand flies (Figure 7I), by 48 hours all granulocytes found in the dermis and hypodermis were eosinophils (Figure 7F and J).

### Changes in the BluePort parenchyma during an ongoing immune response to sand fly bites

**Figure 8:**
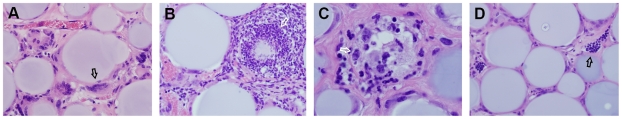
Histopathological changes in the parenchyma of a BluePort adjacent to sand fly bite-induced dermal eosinophilia. Images represent inflammatory changes observed 24 hours after exposure, for a second time, to the bite of sand flies on BluePort-associated skin. Arrows indicate: foreign-body giant cells (A), attack and destruction of CBa-beads by neutrophils (B) and macrophages (C), and lymphocytic infiltration of the BluePort parenchyma (D). Hematoxylin-eosin stain, 1000x magnification.

In addition to the extensive dermal infiltration around sand fly bite sites in immune mice, several features indicative of immune-defense activation were observed scattered in the parenchyma of the associated BluePort, including: 1) the formation of clusters of foreign-body giant cells (Figure 8A), 2) attack and selective destruction of some CBa-beads by neutrophils (Figure 8B), and macrophages (Figure 8C), and 3) infiltration by lymphocytes (Figure 8D). Interestingly, most of these foci of inflammatory- and immune-reactivation were not found in close proximity to the dermal segments where the reactions triggered by sand fly bites were taking place, an indication that a long-range activation mechanism must be responsible for this phenomenon. One possibility is that the newly formed venules and capillaries inside the BluePort were integrated in a portal-like arrangement with the blood supply network of the skin, allowing for cytokines, chemokines and other soluble molecules synthesized at the bite site to be transported into the BluePort parenchyma, following a tissue perfusion dynamics similar to that of neuropeptides and hormones in the hypothalamus/pituitary axis [37].

### Lectin-blot analysis of *L. longipalpis* salivary gland lysates

**Figure 9:**
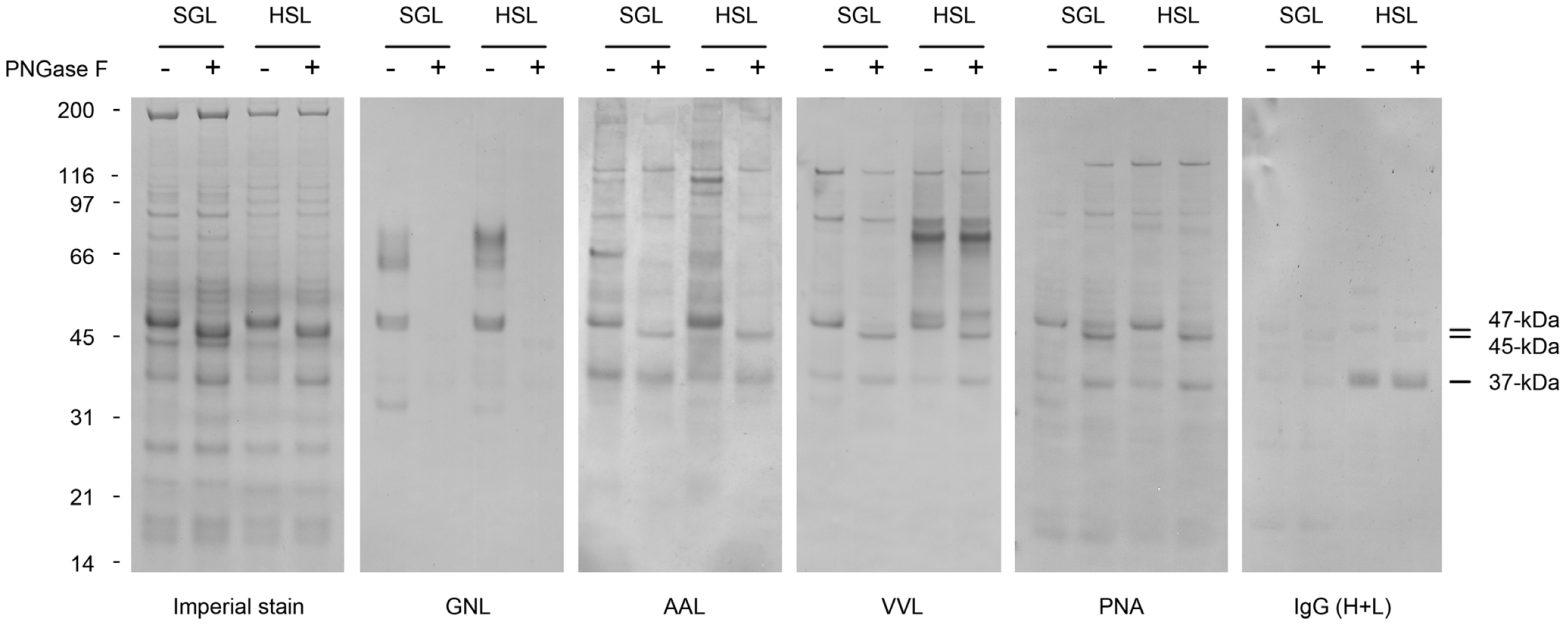
Lectin-blot and immuno-blot analysis of *L. longipalpis* salivary glycoproteins. Effect of enzymatic deglycosylation with PNGase F on two sources of sand fly salivary glycoproteins, SGL and HSL. Samples were stained for protein composition profile (Imperial), probed for the presence of N-linked glycans (GNL, AAL) and O-linked glycans (VVL, PNA), or probed for reactivity with IgG antibodies of animals exposed to the bite of sand flies on BluePort-associated skin. Arrows indicate the main antigenic salivary glycoproteins.

Two of the best characterized inductors of Th2-type immune responses and tissue eosinophilia, Lacto-N-fucopentaose III and LewisX, are saccharidic in nature [38], [39], raising the question of whether glycans attached to salivary proteins play a role in the recruitment of eosinophils at sand fly bite sites. This possibility is supported by evidence indicating that insect-derived glycans are involved in the etiopathogenesis of allergic reactions to insect bites and stings [40], [41]. As an initial approach to explore this possibility, we conducted a lectin-blot analysis of two lysates (SGL and HSL) enriched in sand fly salivary proteins, using biotinylated lectins that specifically recognize N-linked glycans (GNL, AAL) and O-linked glycans (VVL, PNA) (Figure 9). After treatment with PNGase-F, an endoglycosidase that specifically removes N-linked glycans attached to asparagine residues of proteins [42], the electrophoretic mobility of the most abundant glycoprotein in the lysates shifted from 47-kDa to 45-kDa, indicating the presence of an N-linked glycan of approximately 2-kDa (Figure 9A). This was corroborated by showing that the reactivity of the 47-kDa band with GNL, a lectin specific for mannose-rich N-linked glycans, disappeared after deglycosylation with PNGase-F (Figure 9B). The fact that the 45-kDa band was still recognized by a lectin (AAL) specific for fucosylated N-linked glycans (Figure 9C) suggests that two different N-linked glycans are attached covalently to the 47-kDa glycoprotein, a PNGase F-susceptible mannose-rich glycan and a PNGase F-resistant fucosylated N-linked glycan. This interpretation is consistent with evidence indicating that PNGase-F cannot remove N-linked glycans containing core alpha1-3fucose residues [43], one of the determinants recognized by the AAL-binding site [44]. Given that the partially deglycosylated 45-kDa glycoprotein is recognized by two lectins that are specific for O-linked glycans (Figures 9D and E), an alternative explanation is also possible, that AAL reacts with fucosylated O-linked glycans similar to those described in *Schistosoma mansoni* glycoproteins [45]. One additional 37-kDa glycoprotein was found in the sand fly lysates with a similar lectin-binding profile (GNL-, AAL+, VVL+, PNA+) to that of the partially deglycosylated 45-kDa glycoprotein. Interestingly, IgG antibodies against these glycoproteins were found in the sera of mice exposed to the bite of sand flies on BluePort-associated skin (Figure 9F).

## Discussion

In this study we describe a novel tissue compartment induced in the subcutaneous tissue of mice by the injection of CBa-beads and how it can be used to study the dermal response to the bite of *L. longipalpis* sand flies, one of the arthropod vectors of *Leishmania* parasites in the New World. CBa-beads have been used extensively in affinity chromatography protocols to purify, or remove, proteins from complex biological fluids [46], [47], but have not been used before as tissue scaffolds or as a vaccination platform, two of the potential applications that derive from this study. The complex chemistry of Cibacron blue allows it to bind to many different proteins including one, albumin, with the potential to explain the peculiar fate of CBa-beads in mouse tissues. Given the absence of receptors for albumin on the surface of cells of the innate defense system, albumin-covered surfaces might be rendered invisible to the mouse defense systems, and in the absence of danger signals, integrated into the connective tissue of mice. This interpretation is supported by data linking affinity for albumin with the biocompatibility of biomaterials used in variety of medical applications (dialysis, vascular grafting, tissue scaffolding, etc) [48], and the theoretical model describing albumin and other non-defense proteins as tissue-reactivity silencers [49]. An alternative explanation for the fact that CBa-beads are integrated, rather than rejected, might be that Cibacron Blue deliver signals that deactivate, or alternatively activate, macrophages recruited during the chronic inflammatory response to the beads [50]. Under this scenario, the intimate contact of macrophages with CBa-beads might be expected to produce a protracted anti-inflammatory state that allows for the maturation phase of the wound healing process to proceed [36].

The skin overlying a BluePort is of normal appearance and can be used as an access window for the BluePort-resident macrophages to take up and process antigens, including those delivered by sand flies during hematophagy. As shown on Figure 3, the induction of BluePort formation increases the density of professional antigen-presenting cells (F4/80+ macrophages) on the skin segment where sand flies were allowed to blood-feed. It remains to be shown whether these macrophages actually take up, process and presents sand fly salivary proteins to cells of the adaptive immune system. It also remains to be shown whether dendritic cells, another lineage of antigen-presenting cells, are recruited to this tissue compartment.

The tremendous difference in the acute inflammatory reaction seen on BluePort-associated skin of mice exposed once, or twice, to the bite of *L. longipalpis* sand flies, suggests that an immune response to the first bite was enough to significantly modify the intensity and character of the response to the second exposure. A shift in the inflammatory response at the bite site, from a predominantly neutrophilic infiltrate to a predominantly eosinophilic infiltrate, was the main histological feature associated with this response. Whether this shift in the inflammatory response represents a *bona fide* surrogate of protection remains to be determined. In contrast to the well characterized role that eosinophils play in the immunobiology of schistosomiasis and filariasis [51], [52], their role in the immunobiology of leishmaniasis has not been studied as extensively. While it has been demonstrated that eosinophils can phagocytize and destroy *Leishmania* parasites *in vitro* [53] and *in vivo* [54], and that they can be found in skin lesions [55], [56], [57], it is unclear how this translates into a resistance mechanism to parasite transmission by infected sand flies. This is in part a reflection of a systematic artifact introduced in the design of experiments to study interaction of *Leishmania* parasites with their vertebrate host, ie, the absence of sand fly saliva in the inoculate. As a result of this artifact, the conclusion regarding the protective anti-*Leishmania* effect associated with Th1-type immune responses [58], may apply only for artifactual models of *Leishmania* infections where parasites are injected in absence of sand fly saliva. Under natural conditions of transmission (infective inoculum containing both parasites and vector saliva) it is possible to envision a Th2-driven and eosinophil-mediated immune response that delivers protective anti-*Leishmania* effects. A high eosinophil/neutrophil ratio in the granulocyte infiltrate at the bite site could be associated with protection if cytotoxic cationic proteins released during eosinophil degranulation [59], or eosinophil-mediated phagocytosis [60], destroy the parasites before they use neutrophils as a Trojan horse to reach the intracellular compartment of deactivated macrophages [61]. A major problem with this theoretical protection mechanism is that it needs to eliminate all parasites present in the infectious inoculum, otherwise an exacerbated form of the disease can be anticipated once surviving parasites infect deactivated macrophages. This is because their growth would proceed unhindered in the presence of the Th2-type cytokines that are necessary to promote the recruitment of eosinophils into tissues [59], [60]. From a vaccine development perspective this situation represents a conundrum: On the one hand, a Th2-driven vaccine targeting sand fly salivary proteins would be required to be 100% effective in eliminating the inoculated parasites at the sand fly bite site, and on the other hand, a Th1-driven vaccine targeting the same salivary proteins might mitigate the naturally acquired anti-salivary immunity developed as a result of chronic exposure to sand fly saliva. The BluePort system might be an ideal tool to clarify this problem because their resident macrophages can be readily infected with *Leishmania* parasites (data not shown).

The identification of salivary molecules and epitopes capable of promoting the recruitment of eosinophils at the bite site is an important element of efforts to understand the molecular basis of pathogen transmission by hematophagous arthropods. One of the best characterized *L. longipalpis* salivary proteins (maxadilan) has been shown to induce Th2-biased immune responses [17], [62], but it is unknown whether it promotes dermal eosinophilia at the bite site. While it remains to be shown whether any of the glycans attached to *L. longipalpis* salivary proteins promotes dermal eosinophilia, it is intriguing that AAL, a lectin that recognizes *L. longipalpis* salivary glycoproteins, also recognize immunomodulatory glycans expressed in the eggshells of a pathogen, *S. mansoni* [44], that induces strong eosinophilic responses in the tissues of infected animals. The potential for structural similarity in glycans synthesized by arthropods and helminths illustrate the need to study the evolution of the Golgi system in metazoans, and a comparative analysis of the repertoire of glycans that each species can (and cannot) synthesize. It has already been shown that structural similarity in fucosylated N-linked glycans synthesized by plants, insects and nematodes define one of the main cross-reactive epitopes recognized by antibodies of patients with allergies to foodstuff, pollen, insect bites and stings [40], [41]. The high immunogenicity of these glycans, and the cross-reactivity of the antibodies they induce, raises one possibility of great significance in the immunobiology of *Leishmania* transmission by infected sand flies; that anti-salivary immunity may derive not only from chronic exposure to the bite of sand flies, but also from exposure to similar glycans synthesized by plants, helminths or non-hematophagous insects.

It is apparent that the BluePort is a versatile tissue platform that can be used to study the immunobiology of diseases transmitted by hematophagous arthropods, and to facilitate the interaction of salivary proteins with professional antigen-presenting cells in order to induce transmission-blocking anti-salivary immunity. Three anatomical and functional features of the BluePort compartment makes it an ideal tool to dissect complex immunobiological processes: 1) high density of antigen-presenting cells, 2) prominent blood supply network that provides nourishment and access for infiltrating cells of the innate and adaptive immune systems, and 3) accessibility for the delivery of antigens or pathogens, and retrieval of biological samples. A lymph node-like structure with these characteristics represent a novel technology with the potential of transforming the way we study arthropod-borne diseases and develop vaccines to prevent them.

## Materials and Methods

### Injection of Cibacron Blue-agarose (CBa) beads

For induction of BluePort formation, 200 µl of 50% slurry of Cibacron Blue-agarose beads (Sigma, St. Louis, MO) equilibrated in sterile PBS was injected in the subcutaneous tissue of 8–10 week old BALB/c female mice. The procedure was performed using syringes fitted with 23-gauge needles on the abdominal wall of animals anesthetized with ketamine 75 mg/kg and xylazine 15 mg/kg. Animals were sacrificed 6, 24, 48, 72, 96 hours and 15, 30, 60, 90 and 120 days after injection of the CBa-beads. Tissue samples were fixed in 10% formalin, processed routinely, sectioned at 5 microns, and stained with H&E (Premier laboratories, Boulder, CO).

### Histopathological analysis of sand fly bite sites

A colony of *L. longipalpis* sand flies (Lapinha cave strain) was reared following previously described methods [63], [64]. Adult females collected 3–5 days after emergence were allowed to feed on the shaved skin of normal mice or on the skin overlying the blue nodule (BluePort) formed 30–35 days after injection of CBa-beads. Anesthetized animals were placed on top of cartons with 50–100 unfed female sand flies for 20–30 minutes. Three groups of animals were exposed to the bite of sand flies: 1) naïve mice, 2) mice pre-exposed once or 30 times to the bite of *L. longipalpis* sand flies on normal skin, and 3) mice pre-exposed once on BluePort-associated skin. Tissue samples were collected for histopathological analysis 24, 48, 72 and 96 hours after exposure.

### Immunohistochemical analysis of BluePort-resident cells

Tissue samples taken 30 days after injection of CBa-beads were processed for detection of markers specific for mouse endothelial cells (CD31), or macrophages (F4/80), following protocols standardized for each antibody (Premier). Tissue samples were embedded in paraffin, sectioned at 4 microns and deparaffinized in two changes of xylene and hydrated to water through a series of alcohol gradients. Two different methods of antigen retrieval were used: incubation in a TRIS/EDTA pH 9.0 target retrieval solution (Dako, Carpinteria, CA) for 20 minutes at 95°C (for CD31 expression), and incubation with Proteinase K (Dako) for 5 minutes at room temperature (for expression of F4/80). To quench any endogenous peroxidase activity the sections were incubated in a 3.0% hydrogen peroxide solution at room temperature for 5 minutes. Serum Free Protein Block (Dako) was used at room temperature for 5 minutes to neutralize any charged molecules on the tissue sections that may cause non specific staining. Working dilution of the primary antibodies: rat anti-mouse CD-31 antibody (Dianova, Hamburg, Germany) and rat anti mouse F4/80 antibody (AbD Serotec, Raleigh, NC), were prepared in antibody diluent (Dako). The negative control sections were incubated with a rat IgG2a isotype solution (AbD Serotec) at the same duration, concentration and temperature as the primary antibody. The primary antibodies were then conjugated with rabbit anti-rat immunoglobulin (Dako) for 30 minutes at room temperature. This secondary antibody was then labeled with Envision+HRP rabbit polymer (Dako) for 30 minutes at room temperature. Staining was developed with a DAB+ chromogen system (Dako) for 5 minutes at room temperature. Counter staining was performed with Automation Hematoxylin for 10 minutes at room temperature. Sections prepared from a mouse tissue xenograph injected with a tumor cell line that expresses CD-31 for vascularity, or with a HT-29 tumor cells line that expresses F4/80, were used as positive controls. Photomicrographs were acquired with an Olympus DP71 camera and associated computer software.

### Preparation of salivary gland lysates

Collecting pure saliva from sand flies is technically challenging, so we used salivary gland lysates, which have been shown by proteomic analysis to be enriched in salivary proteins [65], as source of glycoproteins for lectin-blot analysis. Salivary glands were dissected from 3–5 day old female *L. longipalpis* sand flies and stored in groups of 20 pairs at −80°C until needed. To prepare the salivary gland lysate (SGL), the glands were resuspended in PBS containing a 1∶50 dilution of protease inhibitor cocktail set III, EDTA-free (EMD Chemicals, Gibbstown, NJ) at a ratio of 1 µl of buffer per pair of salivary glands. After incubating at −20°C for 1 hour, the samples were centrifuged at 18,000×g for 10 minutes at 4°C, and the soluble phase containing salivary glycoproteins, stored at −80°C until used. As an alternative to the cumbersome salivary gland dissection process, we devised a method to collect samples enriched in sand fly salivary proteins. It takes advantage of the fact that when the head of a sand fly is pulled from the rest of the body, the salivary glands remain attached to the head. The collection of the head salivary lysate (HSL) was conducted using the same buffer as above at a ratio of 1 µl of buffer per head. After incubating at −20°C for 1 hour, the samples were centrifuged first at 10,000×g for 1 minutes at 4°C to remove the large insoluble heads, and then at 18,000×g for 10 minutes at 4°C to collect the final soluble lysate.

### Lectin-blot and Immuno-blot analysis of salivary gland lysates

Electrophoretic separation of salivary glycoproteins was conducted under reducing conditions using NuPAGE MES SDS running buffer and NuPAGE 4–12% Bis/Tris precast SDS-PAGE gels (Invitrogen, Carlsbad, CA). Imperial protein stain (Thermo Scientific, Rockford, IL) was used to detect protein bands, and broad-range markers (Bio-Rad) to calculate their molecular weight. Each lane was loaded with lysates derived from seven pairs of salivary glands. By running side by side each lysate before and after treatment with PNGase F (New England Biolabs, Ipswich, MA), it was possible to visualize a shift in molecular weight caused by removal of N-linked glycans. The enzymatic reaction was performed following the manufacturer instructions for 2 hours at 37 C, using 10 units of PNGase F for each µl of lysate. For lectin-blot analysis, the separated proteins were first electro-transferred to nitrocellulose membranes (Bio-Rad), blocked with 1% type B bovine skin gelatin (Sigma), and incubated with biotinylated lectins (Vector, Burlingame, CA). *Galanthus nivalis* lectin (GNL) and *Aleuria aurantia* lectin (AAL) were used to detect N-linked glycans, whereas *Vicia villosa* lectin (VVL), and Peanut agglutinin (PNA) were used to detect O-linked glycans. Following incubation of the membranes with a 1 µg/ml dilution of biotinylated lectins for 1 hour at room temperature, the membranes were incubated with a 1 µg/ml dilution of NeutrAvidin (Thermo Scientific) and biotinylated alkaline phosphatase (Vector) for 1 hour at room temperature. The binding reaction was revealed by incubating the membrane in a phosphatase substrate system, BCIP/NBT (KPL, Gaithersburg, MD), for 15–30 minutes at room temperature. For immuno-blot analysis the membranes were incubated with a 1∶100 dilution of sera collected from mice exposed twice on BluePort-associated skin to the bite of *L. longipalpis* sand flies or, as negative control, the sera of non-exposed mice. The membranes were then incubated in a 1∶1,000 dilution of a goat anti-mouse IgG (H+L) alkaline phosphatase conjugate (KPL) for 1 hour at room temperature, followed by an incubation in alkaline phosphatase substrate as above.
